# Forecasting Daily Radiotherapy Patient Volumes in a Tertiary Hospital Using Autoregressive Integrated Moving Average (ARIMA) Models

**DOI:** 10.7759/cureus.72752

**Published:** 2024-10-31

**Authors:** Thanarpan Peerawong, Chaichulee Chaichulee, Pasuree Sangsupawanich

**Affiliations:** 1 Department of Clinical Research and Medical Data Science, Faculty of Medicine, Prince of Songkla University, Songkhla, THA; 2 Department of Radiology, Faculty of Medicine, Prince of Songkla University, Songkhla, THA; 3 Department of Biomedical Sciences and Biomedical Engineering, Faculty of Medicine, Prince of Songkla University, Songkhla, THA

**Keywords:** automl, autoregressive integrated moving average (arima), healthcare resource management, radiotherapy demand, time series forecasting

## Abstract

Purpose: The purpose is to predict the volume of patients treated daily with radiotherapy using the autoregressive integrated moving average (ARIMA) model.

Methods: In this retrospective study, data from the billing records detailing daily radiotherapy treatment sessions were extracted from the Hospital Information System and analyzed. The study included all patients treated from January 2004 to December 2022. The analysis was divided into two parts: First, the data were summarized using descriptive statistics. Second, time series forecasting with the implementation of an ARIMA model for estimating patient volumes. For the ARIMA modeling process, the Akaike Information Criterion (AIC) was used for classical model optimization. The Mean Absolute Percentage Error (MAPE) was used for evaluating between different models. Residual analysis was performed in each model using the Ljung-Box test, Jarque-Bera test, and heteroskedasticity test to identify autocorrelation, normal distribution, and variances that could undermine the reliability of the model.

Results: A total of 895,808 radiotherapy sessions were included in the study. The median number of radiotherapy sessions per day was 181 (150, 205). A clear transition to more modern radiotherapy equipment, particularly the Truebeam accelerator, was observed, indicating a growing dependency on advanced techniques such as volumetric-modulated arc therapy (VMAT), stereotactic body radiation therapy (SBRT), and stereotactic radiosurgery (SRS). The best ARIMA model predicted an increase in demand, projecting an average daily patient volume of 279.40 by 2030.

Conclusion: The study highlights the need for advanced forecasting methodologies in healthcare resource planning and emphasizes the importance of considering environmental and external factors for effective and accurate resource allocation strategies.

## Introduction

Radiotherapy is essential in cancer treatment, serving both curative and palliative intents. According to the International Atomic Energy Agency (IAEA), over half of cancer patients receive some form of radiotherapy in their lifetime [1]. There is a spectrum of techniques ranging from three-dimensional conformal radiotherapy (3D-CRT) to more advanced methods like intensity-modulated radiotherapy (IMRT) and volumetric-modulated arc therapy (VMAT). These sophisticated techniques have shown improved outcomes in specific cancers, such as head, neck, and lung cancers, by minimizing complications [2-5]. However, they demand more resources in terms of time and personnel. For instance, room occupancy time varies from 16 minutes for 3D-CRT to 24 minutes for IMRT, with VMAT averaging 15-20 minutes. More precise methods like stereotactic radiosurgery (SRS) and stereotactic body radiation therapy (SBRT) require longer durations [6-8]. This underscores the need for careful planning and resource management in radiotherapy.

Effective radiotherapy for cancer depends on strategic resource planning. Various metrics are employed to facilitate this, including the World Health Organization's recommendation measure of radiotherapy units per million population, which is useful for global comparisons but has limitations in local contexts. The radiotherapy utilization rate measures the percentage of new cancer patients treated with radiotherapy within a year and is instrumental in identifying unmet needs, though it requires robust epidemiological data for accuracy [9]. The Malthus model, which utilizes comprehensive variables such as cancer registry data and local surveys, is another approach. While being data intensive, it has been applied successfully in regions such as the UK [10,11]. The effectiveness of each model depends on the quality and availability of data, and in contexts with limited data, time-series models may offer further insights.

Forecasting in healthcare is critical for estimating service demand and patient needs, and it can be achieved through qualitative or quantitative methods. Qualitative methods, like the Delphi method, depend on expert opinions, while quantitative techniques, such as time series forecasting (TSF), utilize historical data for future predictions [12]. In cancer care, TSF methods like regression analysis, autoregressive integrated moving average (ARIMA), and machine learning (ML) are frequently employed to forecast cancer incidence [13-18]. In the context of radiotherapy, regression analysis has been particularly used in estimating patient volumes, such as in cervical cancer studies [19]. Then this study aims to apply TSF techniques to predict daily patient volumes in radiotherapy treatments.

## Materials and methods

Patients and study design

The study presents a retrospective analysis conducted in the Radiation Oncology Unit at Songklanagarind Hospital (PSURO), encompassing all radiotherapy patients treated from January 2004 to December 2022. The data for this analysis were obtained from the hospital's information system. When employing the ARIMA model for time series forecasting, a minimum number of 50 data points is typically recommended to ensure a reliable analysis. However, for more robust and accurate results, it is preferable to use a dataset with more than 100 data points. A larger dataset improves the model's ability to better capture and predict trends and patterns related to radiotherapy patient volume [20].

Statistical analysis

The statistical analysis in this study comprised two parts.

Descriptive Statistics

This initial phase involved summarizing and exploring the dataset, providing a foundational understanding of the characteristics and distribution of the data.

Time Series Forecasting

The ARIMA model is used and the three main components: autoregressive (AR), integrated (I), and moving average (MA) were analyzed to understand the model in detail. ARIMA modeling assumes that the data is stationary and that the residuals are white noise.

First, the stationarity of the dataset was assessed. When visualizing the data, a trend was identified and the stationarity was assessed using the Augmented Dickey-Fuller (ADF) test, often referred to as the “adfuller.” The significance was adequate to achieve stationarity. The AR (p) component of time series models captures the relationship between an observation and its preceding values. The lags represent the temporal intervals between an observation and its past occurrences. The appropriate number of lags, or “p,” is determined using the partial autocorrelation function (PACF). In particular, it evaluates the direct influence of past observations on the current observation. The “cut-off” point at which the PACF values effectively drop to zero. The MA (q) component emphasizes the integration of error terms from previous observations into the model. The autocorrelation function (ACF) is utilized as a diagnostic tool to determine the order “q” of an MA model in time-series analysis. The ACF plot shows how it “cuts off” or effectively drops to zero. If the ACF values reach zero after “q” lags, this indicates that an MA(q) model is appropriate for the data.

When formulating an ARIMA model, the Akaike Information Criterion (AIC) and the Mean Absolute Percentage Error (MAPE) were used for evaluation. The lower the AIC value, the better the model. MAPE assesses the predictive accuracy of the model by calculating the average error as a percentage of the actual values; a lower MAPE value means higher accuracy.

After a basis has been established using conventional statistical techniques, AutoML Pycaret is employed to autonomously optimize a variety of model parameters [21]. This step aims to identify models that can outperform the performance of the manually configured ARIMA model. The critical process involves comparing MAPE values and residual analysis of the models generated by AutoML with those of the ARIMA model.

For the evaluation of residuals, in the time series analysis, the Ljung-Box test is frequently utilized to identify autocorrelation. If the test yields a significant result, this may indicate that the model has not adequately captured the intrinsic structure of the time series. The Jarque-Bera test is applied to determine the normal distribution of residuals and whether the residuals are normally distributed. A significant outcome in this test suggests a deviation from the normal distribution assumption. For the residuals. In addition, to these tests, heteroskedasticity tests are conducted to check whether the residuals have unequal variances, a condition that can undermine the reliability of the model.

## Results

From January 2004 to December 2022, PSURO completed 895,808 radiotherapy sessions, with a median of 181 (150-205) daily treatments, as shown in Table 1. The unit operated seven radiotherapy machines, including two cobalt-based units, Cobalt1 (Theratron 780C, MDS Canada Inc., Canada) and Cobalt2 (Theratron Phoenix, Best Theratronics, Canada), with median daily usage rates of 12 (9-15) and 52 (39-60) sessions, respectively. The linear accelerators, Linac IX and Clinac 2100C (Varian Medical Systems, USA), were more frequently used, with median daily sessions of 103 (90-103) and 84 (90-113), respectively. The Truebeam STX (Varian Medical Systems), designed for advanced therapies, recorded a median daily usage of 28 (10-51) sessions.

**Table 1:** Machine and number of fractions

Machine	Start operation date	Stop operation date	Total fractions	Fraction per day (median (IQR)
Cobalt1	Operated	28/10/2004	2,689	12(9,15)
Cobalt2	Operated	30/12/2011	99,439	52(39,60)
Linac	Operated	1/06/2012	183,082	84(74,93)
MLC	22/02/2006	25/10/2021	111,886	30(13,45)
Unique	6/5/2013	Present	153,452	66(50,76)
IX	21/11/2012	Present	271,021	103(90,113)
Truebeam	15/5/2013	Present	74,239	28(10,51)

**Figure 1:**
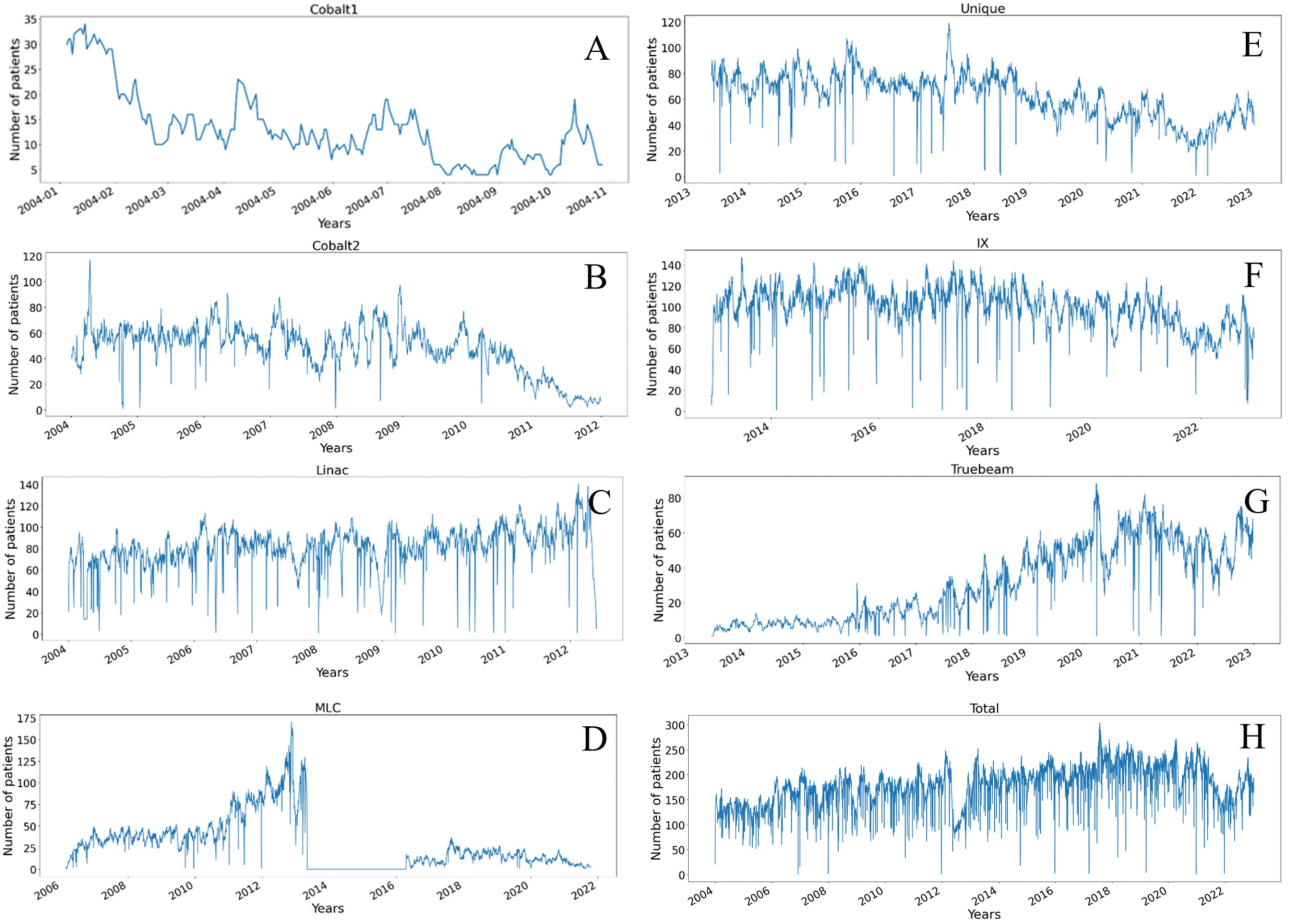
Daily number of patients treated with radiotherapy from 2004 to 2022 (A) Cobalt1 machine. (B) Cobalt2 machine. (C) Linac machine. (D) MLC machine. (E) Unique machine. (F) IX machine. (G) Truebeam machine. (H) Total number of patients.

In 2004, the PSURO operated three key treatment units. During this time the older Cobalt1 unit was decommissioned and a single energy linear accelerator with a multileaf collimator (Linac 6EX MLC: MLC) was installed. Another machine, the high-energy linear accelerator, Clinac, is notable for its versatility, providing multiple photon energies (6 and 10 MV) and a variety of electron energies. This range enables customization for different cancer types and intricate treatment techniques, making it a popular choice for a wide array of clinical applications. In contrast, the Cobalt machine operates at a single energy level of 1.25 MeV. The Cobalt machine's limited adaptability can pose challenges in achieving complex dose distributions or deep tissue penetration. As a result, over the past decade, Clinac has experienced a steady increase in usage, affirming its status as a technologically advanced treatment option. Meanwhile, the usage of the Cobalt2 unit has been declining, as illustrated in Figure 1C. This pattern reflects the shifting preference towards more adaptable and sophisticated treatment modalities in radiation oncology.

The MLC unit played an important role in 2012 and 2013, a transitional period for the facility marked by the new installation of three other radiotherapy machines which replaced Clinac and Cobolt2 units. During this time, the MLC unit accommodated an increased patient load, as evidenced by a surge in its utilization. This heightened usage, as depicted in Figure 1D, ceased by mid-2013, indicating the end of a significant transitional phase in the facility's capabilities. A brief reactivation of the MLC unit occurred in mid-2016 to alleviate the workload on other units. However, this effort was less effective due to the MLC's inherent limitations compared to more advanced units. The limited impact of this reactivation underscored the facility's growing dependence on more sophisticated equipment, such as the Linac Unique (Unique) and IX units, to meet the complex demands of modern radiotherapy.

As Figures 1A-1H illustrate the period between 2013 and 2022 maintained a relatively small increase in the number of treatment fractions per day. A detailed analysis reveals a significant increase in the utilization of Truebeam machines, indicating a shift towards advanced techniques. In contrast, there has been a notable decrease in the usage of the IX and Unique models. This trend signifies a strategic move towards more technologically sophisticated treatment methods in the field of radiotherapy.

The onset of the COVID-19 pandemic toward the end of 2019 initially had no impact on the daily fraction numbers at our facility. However, the introduction of strict lockdown measures in mid-April 2020, including inter-provincial travel restrictions and a curfew, led to a marked decrease in daily treatment fractions. This downturn was largely due to logistical challenges that hindered patients' access to treatment. In response, the facility strategically shifted towards hypofractionation radiotherapy schemes to minimize patient visits and manage these challenges effectively. This shift resulted in a noticeable decrease in daily fraction numbers.

Time series manipulation and consecutive imputation

We processed daily patient data from business days to generate monthly averages, aiming to reduce noise. During this analysis, two periods of system instability were identified: the first occurred between 2012 and 2013, coinciding with the installation of three new radiotherapy machines, and the second in 2020, due to the disruptions caused by the COVID-19 pandemic.

**Figure 2:**
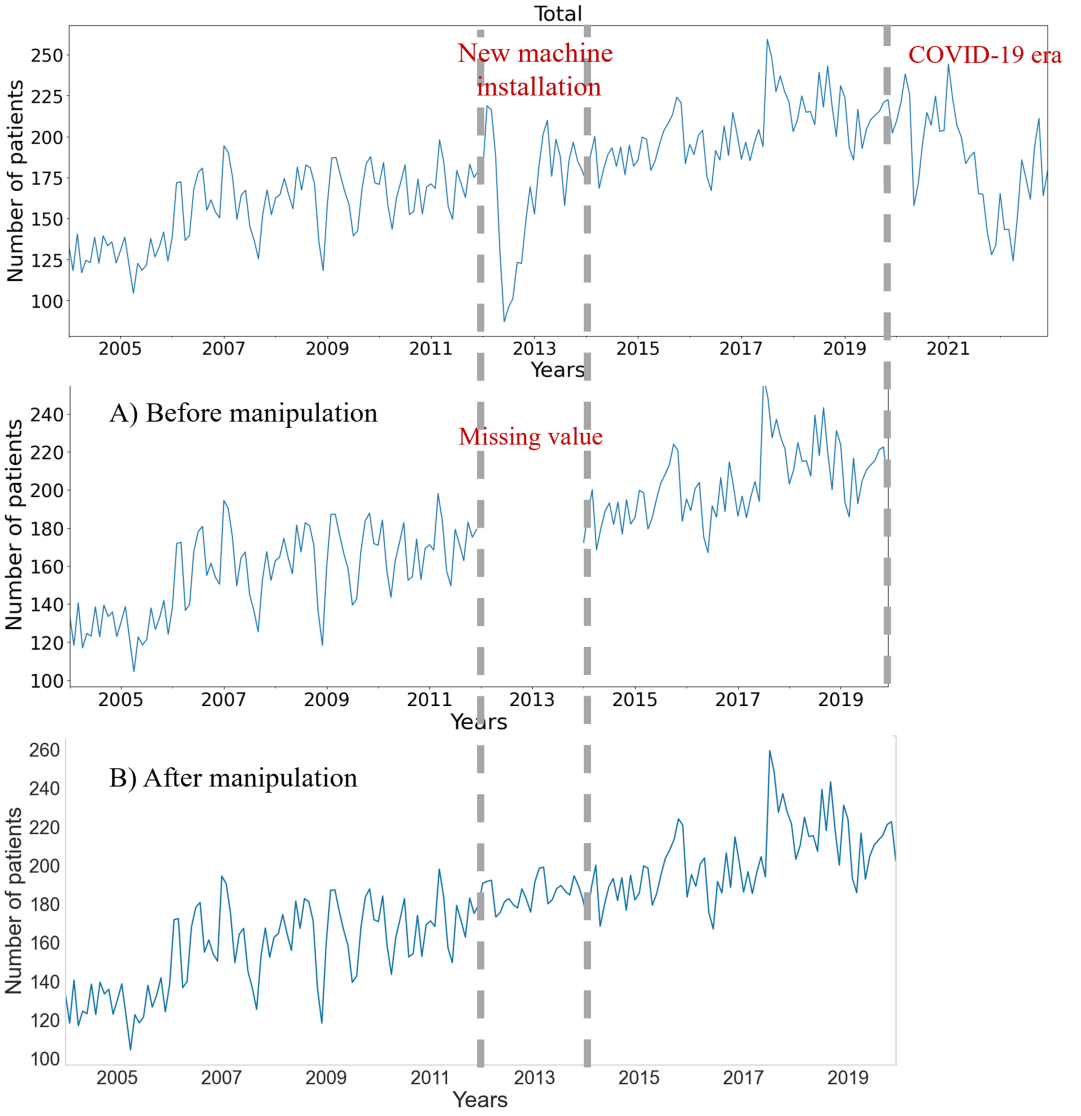
Daily number of patients treated with radiotherapy before and after manipulation

To address data gaps for 2012-2013, we used ARIMA imputation, leveraging historical data from 2004 to account for trends and seasonality. Initially, we visualized the data to identify trends (Figure 2). After applying a degree of differencing, the ADF test confirmed stationarity. The initial parameters for the AR and MA components were set at p = 0 and q = 2, respectively. To account for seasonality, we applied a 12-month difference. The optimal ARIMA model for imputation was determined to be ARIMA(0,1,2)(0,1,1,12), which had a MAPE of 10%.

 Residual analysis showed no autocorrelation, as indicated by the Ljung-Box test (0.01, p = 0.75), and confirmed a normal distribution with the Jarque-Bera test (1.14, p = 0.56). The heteroskedasticity test resulted in a value of 1.05 (p = 0.089). This ARIMA model was then applied to our dataset, with the results illustrated in Figure 2, demonstrating its effectiveness in time series imputation.

Time series forecasting

Upon analyzing the data and performing a time-series analysis, non-stationary behavior was observed. To address this, a single level of differencing (d=1) was applied, achieving stationarity as confirmed by a significant ADF test. The AR and MA components were initially set at p = 4 and q = 3, respectively. The ARIMA(4,1,3) model's performance was evaluated using MAPE, yielding 7.1% for the training set and 9.3% for forecasting. Residual analysis indicated a normal distribution and no autocorrelation. A seasonal component was then incorporated, resulting in the ARIMA(1,1,1)(0,1,1,12) model, which had MAPE of 7.5% for the training set and 7.3% for forecasting. Residuals from this model conformed to statistical assumptions.

Using AutoML for model optimization, the final selected model was ARIMA(0,1,2)(1,0,0,24), based on its superior statistical parameters. This model was deemed the most appropriate for modeling and forecasting patient volume data. The details of the model and residual evaluation are shown in Table 2.

**Table 2:** Comparison model parameter of various ARIMA models for daily radiotherapy fraction ARIMA - autoregressive integrated moving average

Model	MAPE (train)	MAPE (test)	Ljung-Box (p-value)	Jarque-Bera (p-value)	Skew	Kurtosis	Heteroskedasticity (p-value)
ARIMA(4,1,3)	0.071	0.092	0.32 (0.57)	0.09 (0.96)	-0.02	3.11	0.61 (0.08)
ARIMA(1,1,1)(0,1,1,12)	0.075	0.073	0.14 (0.71)	1.82 (0.40)	-0.28	2.98	0.62 (0.10)
ARIMA(1,0,0)(0,1,0,24) (autoML)	0.221	0.091	0.90 (0.34)	0.23 (0.89)	0.10	3.03	0.54 (0.04)
ARIMA(0,1,1)(1,1,0,24) (autoML: tuned-model)	0.088	0.081	8.94 (0.00)	0.10 (0.95)	-0.02	2.87	0.54 (0.04)
ARIMA(0,1,2)(1,0,0,24) ( autoARIMA)	0.072	0.081	0.00 (0.96)	0.28 (0.87)	-0.10	3.08	0.62 (0.09)
ARIMA(0,1,2) (autoARIMA: tuned-model)	0.075	0.090	0.01 (0.91)	0.61 (0.74)	-0.14	2.86	0.66 (0.14)

**Figure 3:**
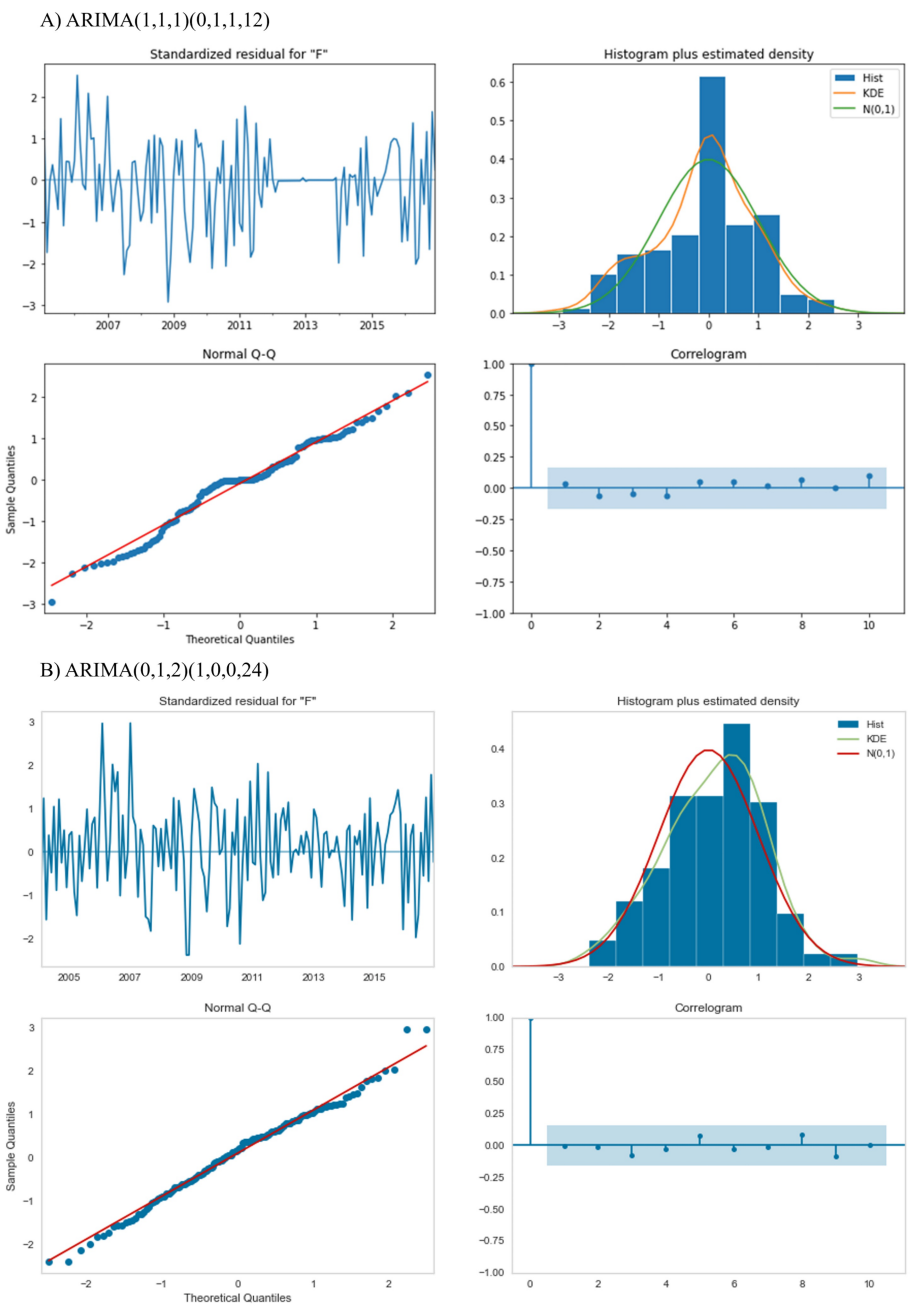
Comparative residual analysis: (A) ARIMA(1,1,1)(0,1,1,12) and (B) ARIMA(0,1,2)(1,0,0,24) ARIMA - autoregressive integrated moving average

**Figure 4:**
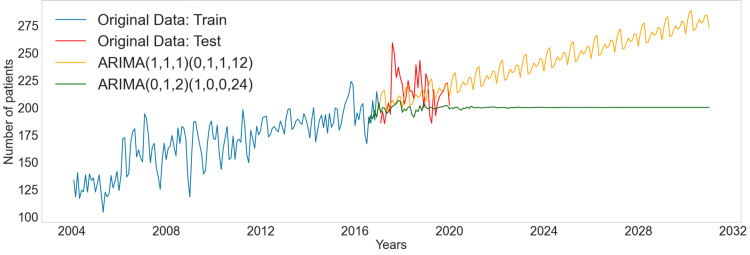
Forecasting plot comparing between ARIMA model ARIMA - autoregressive integrated moving average

In the final evaluation phase, two ARIMA models were compared for forecasting accuracy. While both models demonstrated strong statistical performance, the ARIMA(1,1,1)(0,1,1,12) model showed a more precise fit in the Q-Q plot and a closer adherence to normal distribution (Figures 3A, 3B). However, it exhibited reduced residual distribution during the imputation period. Figure 4 illustrates the forecasting plots, projecting an average patient volume of 279.40 per day for 2030.

Exogenous factors, including the number of radiation oncologists, medical physicists, and external center radiotherapy machines, along with the period of high-technology adoption, were incorporated into the ARIMA(1,1,1)(0,1,1,12) model. Despite this, residual analysis showed no autocorrelation and a normal distribution, but these factors did not significantly influence the model's predictive accuracy. This suggests that while the model's residuals were statistically sound, the exogenous variables had minimal impact on its predictive capability.

## Discussion

The study provides two critical insights. Firstly, the study identifies a clear pattern between the technological advancement of radiotherapy treatment units and their usage. It notes that units with outdated technology experienced a decline in utilization over the observed period. Conversely, treatment units that are equipped with state-of-the-art technology and offer high-performance capabilities have witnessed increased usage. This trend underscores the growing preference for and reliance on technologically advanced equipment in the field of radiotherapy. Secondly, the study forecasts an average of approximately 279.40 patients per day receiving radiotherapy in 2030. This prediction notably surpasses the median daily patient count observed throughout the entire study period. This upward trend in the forecasted patient volume suggests an increasing demand for radiotherapy services in the coming years.

The results of the study indicate that technological progress in radiotherapy has influenced the choice of treatment units and patient throughput overall. Taking Korea as a case study, the increasing adoption of advanced techniques like IMRT is evident, with its usage escalating from 2% in 2011 to 50% in 2019 [22]. Our result, the transition from 3D to modulated beam radiotherapy, was found during 2014-2022. The number of patients treated with the Truebeam machine has increased. However, according to treatment precision, the imaging before each treatment session becomes an important part of treatment [23,24]. While this shift has enhanced the quality of treatments, this could paradoxically lead to a decrease in the number of patients treated daily. These could be critical challenges for healthcare resource planning to avoid potential bottlenecks and ensure efficient patient care in the landscape of radiotherapy.

The onset of the COVID-19 pandemic has influenced healthcare practices, including radiotherapy protocols. To adhere to social distancing management and reduce patient density in healthcare facilities, treatment schedules have been modified, leading to an increased adoption of hypofractionated radiotherapy regimens. In palliative care, the trend is towards single-fraction treatments or a limited number of fractions, reducing the frequency of patient visits to healthcare settings [25]. For curative treatments, such as in breast cancer, there has been a shift in protocols. Breast-conserving radiotherapy now typically involves 26 Gy delivered in five fractions, as opposed to the previous 40 Gy in 15 fractions [26]. Similarly, for locoregional treatments, the protocol has transitioned from 50 Gy in 25 fractions to 40 Gy in 15 fractions [27,28]. These adjustments serve to both comply with social distancing requirements and alleviate the burden on healthcare resources, thus minimizing COVID-19 transmission risks. While it is uncertain if these crisis-induced fractional schedules will become standard practice, they have increased the openness of radiation oncologists to hypofractionated approaches.

The assessment of machine workload and capacity is a critical aspect of radiotherapy management. According to the most accurate ARIMA model in our study, the daily patient load is forecasted to be 279.40 in 2030. With an estimated “door-to-door” time of 12 minutes per patient, this translates to a requirement of 56 service hours per day [6]. To accommodate this demand within a 12-hour operational schedule per machine, about four to five machines would be necessary. However, in an ideal clinical scenario where each patient’s treatment takes 15 to 20 minutes, the total daily service hours needed would increase to between 70 and 94 hours. Under a standard eight-hour clinical day, this would necessitate between nine and 12 machines to maintain optimal patient care levels. On the other hand, the analysis of exogenous variables did not yield significant results. The study was unable to ascertain the impact of treatments conducted outside the institute. Consequently, estimating the decline in patient numbers per additional machine from external sources presents a challenge. This highlights a potential area for further investigation in the field of radiotherapy resource management.

The study is subject to two primary limitations. Firstly, it relies on univariate time series analysis using ARIMA models, which depend on historical data. This approach overlooks potential influencing factors such as machine availability and healthcare policies, possibly impacting forecast accuracy. Additionally, the dataset is derived from billing records, potentially not reflecting the actual demand for radiotherapy. Hospital accreditation data indicate efficient resource optimization, maintaining waiting times within a month, suggesting that the real demand could surpass our estimates.

Secondly, although recent advancements in machine learning and deep learning, including long short-term memory (LSTM) and time series transformer models, have shown improved predictive capabilities in cancer control and forecasting, they pose interpretability challenges [13-18]. Their complexity often results in a “black-box” scenario, especially problematic in healthcare where understanding causative factors is vital for effective policymaking. Future research should strive for a methodology that combines high predictive accuracy with clear, interpretable insights. This balance is essential for translating findings into practical applications in healthcare planning and resource allocation in radiotherapy.

## Conclusions

In conclusion, this study's estimation for healthcare resource planning based on ARIMA models provides an important basis for initial planning. However, it also highlights the need for more comprehensive methodologies. These are required to incorporate environmental and external factors, which are crucial for a more accurate and effective resource allocation strategy in healthcare settings. The study emphasizes the importance of going beyond univariate time-series analysis to encompass a broader range of influences in healthcare planning.
